# Musical Metaverse: vision, opportunities, and challenges

**DOI:** 10.1007/s00779-023-01708-1

**Published:** 2023-01-13

**Authors:** Luca Turchet

**Affiliations:** Department of Information Engineering and Computer Science, University of Trento, Trento, Italy

**Keywords:** Music, Metaverse, Extended reality, Internet of Musical Things, Musical XR, Music industry

## Abstract

The so-called metaverse relates to a vision of a virtual, digital world which is parallel to the real, physical world, where each user owns and interact through his/her own avatar. Music is one of the possible activities that can be conducted in such a space. The “Musical Metaverse” (MM), the metaverse part which is dedicated to musical activities, is currently in its infancy, although is a concept that is constantly evolving and is progressing at a steady pace. However, to the best of the author’s knowledge, as of today an investigation about the opportunities and challenges posed by the MM has not been conducted yet. In this paper, we provide a vision for the MM and discuss what are the opportunities for musical stakeholders offered by current implementations of the MM, as well as we envision those that are likely to occur as the metaverse emerges. We also identify the technical, artistic, ethical, sustainability, and regulatory issues that need to be addressed so for the MM to be created and utilized in efficient, creative, and responsible ways. Given the importance and timeliness of the MM, we believe that a discussion on the related opportunities and concerns is useful to provide developers with guidelines for creating better virtual environments and musical interactions between stakeholders.

## Introduction

Throughout history, the way music has been composed, played, learned, and experienced has constantly evolved on the basis of technological developments and shifting musicians’ and audiences’ preferences. Today, the so-called metaverse is emerging as a new space where musical activities can be conducted. The metaverse relates to a vision of a virtual, digital world which is parallel to the real, physical world, where each user owns and interacts through his/her own avatar [1–5]. Such a vision builds upon a long and rich history of research endeavors in both academy and industry about the creation and study of immersive technologies, gaming platforms, and cyberspaces for social interactions [6].

To date, a definition of the term metaverse within the literature has yet to be agreed upon. However, in this paper, we align with the succinct definition detailed in [3, 4] that considers the metaverse as a virtual environment blending the physical and the digital, facilitated by the convergence between Internet of Things and Extended Reality (XR) technologies. According to the Reality-Virtuality Continuum proposed by Milgram and Kishino [7], XR integrates the digital and the physical to various degrees, ranging from Augmented Reality (AR), to Mixed Reality (MR), to Virtual Reality (VR). Therefore, the metaverse overcomes the temporal and spatial boundaries of physical reality and offers users an immersive experience. It can be considered as the next big step in the evolution of the Internet, where connected users will be able to virtually interact with each other, with the seemingly experience of sharing the same environment. Such a cyberspace will allow an unlimited number of users to work, purchase, sell, collaborate, socialize, create, learn, and play. This vision, however, is currently in its infancy despite the grand fanfare around this concept recently made by big tech giants to promote their envisioned future business lines.

To date, different types of metaverses exist, such as gaming based (e.g., Fortnite, Roblox, Second Life, or Minecraft) or blockchain based (e.g., Decentraland or The Sandbox). Some metaverses (e.g., Horizon by Meta) are completely based on VR technologies and require a VR headset to immerse the user in the virtual world. In general, all these types of metaverse have in common one element, that is, a virtual place where one can interact in real time with the digital environment, as well as with real people (in the form of their avatars). Different technologies, such as XR, 5G/6G, cloud computing, blockchain, digital twins, and artificial intelligence, are converging toward the implementation of the metaverse. Such a convergence is impacting numerous domains, such as video games and business [8], and music is no exception.

The metaverse offers the possibility to build social relationships and participate in or even co-create an entertainment. One of the most popular forms of entertainment is music, and a large number of virtual clubs and virtual concert halls have been built in recent years to cater for this need, where people can gather, meet, make friends, dance, and enjoy both live music and listening to recordings. Different musical activities can be conducted in the metaverse, from composing to performing, to recreational music making, to teaching, to experiencing a virtual live concert. This has implications not only at artistic level, but also at commercial level. For instance, in October 2021, Decentraland (a decentralized virtual social platform powered by the Ethereum blockchain) hosted the world’s first Metaverse Festival, which lasted 4 days and included a lineup of more than 80 high-profile musicians.^1^ The event was free for participants and was even sponsored by important brands.

All these trends are leading to the emergence of what we coin as the “Musical Metaverse” (MM), the metaverse part which is dedicated to musical activities. Whereas the field is rapidly progressing, the MM vision is currently in an embryonal state. The MM is a concept that is constantly evolving and different musical stakeholders are enriching its meaning in their own ways. However, to the best of the author’s knowledge, as of today a comprehensive investigation about the opportunities and challenges posed by the metaverse used for musical activities has not been conducted yet. In this paper, we discuss what are the opportunities for musical stakeholders offered by current implementations of the MM, as well as we envision those that are likely to occur as the metaverse emerges. We also identify the technical, artistic, personal data, and regulatory issues that need to be addressed so for the MM to be created and utilized in efficient, creative, and responsible ways.

The MM is not a novel concept. Already in the past decade, authors discussed the metaverse in its musical flavor especially focusing on the Second Life platform [9]. Nevertheless, in those years, the times were not mature for the metaverse to unfold and be adopted on a large scale: the technology for XR and credible networked interactions was in a primordial stage, the social networks only started to be mass adopted, digital currencies were not widespread, and systems for music broadcasting, streaming, or networked music performances were not optimal.

Today, such technological and non-technological contexts are radically different, and the willingness and need to musically interact online has been accelerated by the recent COVID-19 pandemic [10–13]. Moreover, the metaverse started to attract the attention of the wider masses after recent announcements of social media giants and big technology companies claiming the metaverse as the future of the Internet (e.g., by Mark Zuckerberg CEO of Meta^2^). All these aspects have contributed to a new dawn of the MM and the metaverse in general. Given the importance and timeliness of the MM, we believe that an investigation on the related opportunities and concerns is useful to provide developers with guidelines for creating better virtual environments and musical interactions between stakeholders, as well as researchers with new elements for further discussions.

## Background

### Musical XR

The past two decades have witnessed a rapid growth in the number of academic, artistic, and industrial works at the confluence of XR and music, leading today to an established area of research which can be referred to as “Musical XR” [14]. Various authors have surveyed different aspects of this broad field. A review of virtual reality musical instruments up to 2016 was surveyed by Serafin et al. [15]. Berthaut [16] reviewed 3D interaction techniques and examined how they can be used for musical control. A survey of the emerging field of networked music performances in VR was offered by Loveridge [17]. Atherton and Wang [18] provided an overview of recent musical works in VR, while Çamcı and Hamilton [19] identified research trends in the Musical XR field through a set of workshops focusing on Audio-first VR. However, it is important to acknowledge that Musical XR is not just a twenty-first-century phenomenon, but it is today the evolution of an artistic-technological path that has its roots in seminal works of various practitioners positioned at the confluence of music performance and computer science (e.g., [20] and [21] to name a few).

According to Turchet, Hamilton, and Çamcı [14], Musical XR works can be defined through four main characteristics: (1) existence of virtual elements—these are provided in one or more sensory modalities, and in augmented or fully immersive contexts,(2) spatial persistence—the virtual elements share with the user a persistent three-dimensional space; (3) interactivity—this refers to a wide range of interactions of the user with the virtual/augmented environment, from the simple rendering of the virtual space in accordance to the user’s position and head orientation, to the complex sound-producing manipulation of virtual elements; (4) sonic organization—the way the auditory elements and interaction with them are organized is a fundamental aspect of the conceptual and/or technical implementation of a Musical XR system.

The use of XR technologies in musical activities represents a paradigm shift as they disrupt traditional notions of musical interaction by enabling performers and audiences to interact musically with virtual/augmented objects, agents, and environments. A large variety of musical activities have been investigated using the XR medium: systems have been devised for supporting composition (e.g., [22–24]), education (e.g., [25, 26]), performance (e.g., [27–29]), entertainment (e.g., [30–32]), and sound engineering (e.g., [33–36]. Moreover, various software and hardware tools have been devised to support the development of Musical XR systems (e.g., [37–39, 39]), along with design and analysis frameworks [41], while different studies have investigated the perception in Musical XR settings (e.g., [42, 43]).

### Internet of Musical Things

The Internet of Musical Things (IoMusT) is a branch of the Internet of Things (IoT) that specifically targets the musical domain [44]. It relates to the network of Musical Things, which are objects serving a musical purpose. These include smart musical instruments [45] and wearable devices for performers and audiences [46]. In the context of the MM, XR headsets used for musical applications in networked settings are Musical Things in their own right.

The IoMusT networking infrastructure enables multidirectional communication between both co-located and remoted Musical Things, leading to ecosystems of machines and human stakeholders (including performers, amateur musicians, audience members, music students and teachers, studio producers, labels, publishers, and sound engineers). Several IoMusT applications require a fast exchange of musical content between geographically displaced musicians interacting over the network. Such requirement is not necessary in the vast majority of IoT applications [47]. Therefore, an important factor distinguishing the IoMusT from the IoT is latency, which must be kept constantly below 30 ms to support realistic musical interactions enabling musicians to play together over the network [48].

The paradigm of IoMusT is paving the way for a world where many Musical Things will be connected and will interact with their user and environment to collect information and automate certain tasks. Moreover, the sensors embedded in Musical Things provide users with interactive musical experiences that bridge the MM and the real world. As a consequence, the IoMusT is expected to play a vital role in network infrastructure of the MM. Nevertheless, as highlighted in Loveridge [17] and Turchet et al. [14], to date real-time musical interactions in networked Musical XR environments is a rather unexplored area, with only a few studies investigating collaborative music making in XR [49, 50] and even less on interactions mediated by the network [51, 52].

### Blockchain, NFTs, and the music industry

The blockchain technology relates to a decentralized graph of interconnected data units called blocks [53]. It is essentially a distributed ledger (i.e., database) that is not maintained by a single individual but shared among independent nodes of a computer network. The blocks along the chain record the transactions of the distributed ledger digitally in chronological order. Each block points to its previous block along the chain using the hash of the previous block’s header. The key feature of the blockchain technology is that it offers its users a way to carry out transactions with another person or entity in a peer-to-peer manner without requiring a centralized authority or having to rely on third parties. At the same time, it ensures the confidentiality and integrity of the exchanged data using cryptographic mechanisms.

An important aspect of blockchain technology is that of smart contracts [54], which are lines of code that self-execute whenever a certain condition occurs. Devices are allowed to call public code functions contained in a smart contract. Such functions can trigger events, while applications can listen for them to properly react to the triggered event. In order to change the state of the contract, it is necessary to publish a transaction in the blockchain.

In the past few years, several authors have investigated the potential applications of blockchain technology in the recording music industry, identifying different use cases [55–62]. One of the most relevant use cases consists of the creation of a networked database of copyright ownership that would help solving issues with music licensing. To own products via ownership rights and benefit from royalty distribution is today a huge challenge for the music industry. Ownership rights are required to monetize digital music products. Blockchain and smart contracts could be utilized to generate a comprehensive and accurate decentralized database of music rights. Such a database would include all the information distributed in various proprietary databases, and thanks to the blockchain all the data would be automatically and instantly updated, and made available to every user.

A second use case concerns the management of royalties by means of smart contracts. Nowadays, royalty payments to artists are affected by a generalized slowness (they typically occur a few times a year), but via smart contracts they can occur immediately after the consumption of a musical piece. Meantime, the ledger can provide a transparent information about the artists’ royalties as well as real-time distributions to all the labels involved. The payments can be made via digital currency according to the terms of the smart contracts. This includes micropayments, leading to lower transaction costs and quicker times.

A third use case relates to the solution of the issues of transparency within the value chain. Today, it is rather difficult for artists to assess the level of efficiency with which payments are processed by record labels, collective management organizations, or publishers; because of the aforementioned copyright information problems, often a non-negligible amount of revenues does not reach the artists, but it ends up in black boxes in which it is not possible to identify in an accurate way who are the rightful owners of royalty revenue.

However, the use cases discussed above only concern issues in the recording music industry, which is only one of the industries forming the music sector [63]. In the context of the metaverse and blockchain technology, NFTs have emerged as a means to directly support musicians in various musical activities, from composition to live music streaming, to merchandising. NFTs stand for “Non Fungible Tokens” and are the digital properties underlying the metaverse [64, 65]. They are powered by blockchain technology and refer to digital assets that are represented by tokens. A token is a string of text which is encoded on a shared accounting ledger that keeps track of who owns what. Thanks to the underlying blockchain technology, anyone can recognize the ownership of a digital asset, without knowing or trusting the owner. NTFs are characterized by their uniqueness, i.e., there cannot be two identical NFTs. Moreover, NTFs can be programmed via smart contracts to execute actions in an automatic way (e.g., a musician can receive royalties every time a buyer of the tokenized digital asset resells it to somebody else) [66–69].

Today, the metaverse is characterized by a virtual marketplace, driven by NFT trading platforms, which facilitates peer-to-peer trading of digital assets. Such assets have included also music and music-related items. In general, in the past 2 years, NFTs have grabbed the attention of several musicians, both emergent and established, who sold tokenized versions of their tracks or virtual and real-world merchandise (e.g., concert tickets). This has enabled them to increase their revenues without relying to central authorities or third parties, such as record labels, agents, lawyers, and distributors. Moreover, this has allowed musicians to deliver new experiences to their audiences. Furthermore, NFTs have enabled the fans to directly support their preferred artists thanks to crowdfunding and by doing so they also get the unprecedented ownership of part or the whole digital asset sold. In some cases, owning a digital asset even entails rights on its governance, which is a completely new experience for a fan. All this puts a lot of power back in the hands of musicians who now have other ways to monetize their creative efforts or other forms of digital merchandise.

### Digital twins

According to IBM,^3^ “Digital twins are a virtual representation of an object or system that spans its lifecycle, is updated from real-time data, and uses simulation, machine learning, and reasoning to help decision-making.” Digital twins are virtual entities created to reflect physical entities, such as objects, people, and systems [70]. Such virtual entities reflect the properties of their real-world counterparts, including the entity’s physical appearance, behavior, and performance. They are essentially clones of real-world entities, which enable data to be seamlessly transmitted between the physical and virtual worlds. Such digital representations of real-world entities are synchronized with the real world by means of sensors that detect information as well as bi-directional Internet communication. Any change occurring in the real-world entity is reflected in the twin and controls can be sent from the twin to the physical entity. Therefore, digital twins are placed at the intersection of the physical and the virtual world. The capabilities of digital twins offer novel opportunities for a wide variety of applications, including monitoring, simulation, analysis, optimization, or prediction. Moreover, various research efforts are ongoing to integrate digital twins with blockchain technology [71].

All these features make the digital twins an essential component of the envisioned metaverse. Indeed, the metaverse is not solely a platform where to perform individual, collaborative, or business activities. It can also be the place where to simulate the physical world inside a digital world that can be experienced via XR technologies to monitor and optimize the physical world.

Given their features, digital twins can be the cornerstone of the MM, where digital and physical musical entities will behave in a similar fashion. Recreating via this technology musical stakeholders, devices, and venues could impact a variety of musical activities, providing new processes and services. In particular, the creation of digital twins for the MM could leverage the results of several decades of research in various domains of music technology, including virtual-analog DSP methods to simulate the sound and control of real musical instruments [72] as well as virtual acoustic methods for the recreation of the acoustics of the venue where the physical entity is placed [73].

## A Musical Metaverse vision

After having surveyed the metaverse field and the technologies related to its musical counterpart, we attempt to provide a discussion about what could be the MM. First, we propose a definition: *the Musical Metaverse is an interoperable persistent network of multiuser environments merging physical reality with digital reality, which serve a musical purpose. It is based on the convergence of Musical XR and IoMusT technologies that enable multisensory, networked musical interactions between musical stakeholders, as well as between such stakeholders and Musical XR environments and objects.*

This definition is based on an analysis of the various definitions for the metaverse that are available in the literature (e.g., [3, 4, 8, 74, 75] and on their adaptation to the musical domain. Such an analysis identified common attributes of the metaverse, such as the persistence of identity and objects, a set of environments shared among users, the use of avatars, synchronization, being three-dimensional, interoperability, and a user experience that is interactive, immersive, and social. Of course, the proposed definition is broad and may evolve as the metaverse continues to be built and used.

**Fig. 1:**
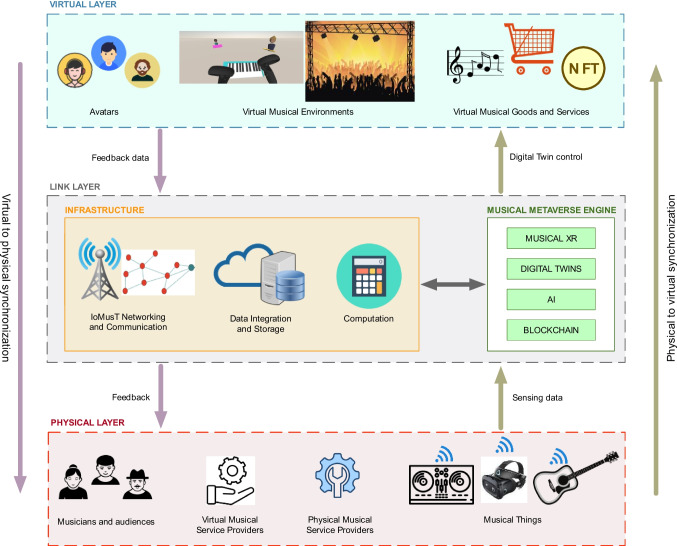
Framework of the Musical Metaverse, which consists of a physical layer, a link layer, and a virtual layer

Second, we propose a framework depicting the technological architecture of the MM, which is based on three layers: physical, link, and virtual (see Fig. 1). This framework is partly inspired by other frameworks recently proposed in the literature for the general metaverse field [76, 77].

### Physical layer

In the physical layer, data about the real musical stakeholders (e.g., musicians’ performative actions, audiences’ gestures and voices) and real environments (e.g., concert venues, recording studios) are collected through Musical Things, which embed intelligent sensing technologies and wireless connectivity. Each musical stakeholder in the physical layer controls components (e.g., Musical Things) that influence the virtual layer. In turn, the virtual layer may provide feedback to the physical layer. The key musical stakeholders are as follows:

- *Users:* these are musicians (e.g., composers, music students and teachers, audio engineers) or audience members, who experience the virtual worlds as avatars via XR equipment such as HMD and XR-based musical instruments. They conduct musical activities (e.g., performing, teaching music, attending concerts) within the virtual worlds and can socially interact between each other.
- *Virtual musical service providers:* these are actors that contribute with content to the virtual worlds of the MM. In the same way today music or video content is created by users on music video sharing platforms (e.g., Soundcloud, YouTube), the MM is envisioned to be enriched with user-generated content that includes music, virtual musical objects and environments, and musical applications. Such a content can be created, traded, and consumed in the MM. In this category of stakeholders may fall also record labels and publishers, as well as rights societies.
- *Physical musical service providers:* these stakeholders develop, maintain, and manage the physical infrastructure that supports the MM engine and respond to transaction requests that originate from the MM. This includes the operations of communication and computation resources or logistics services for the delivery of physical goods transacted in the Metaverse. Such providers may include Musical Things manufacturers and IoMusT service providers, music venues, as well as distributors and other actors in the music industry supply chain who are responsible for logistics or physical products (artists merchandise, CDs, vinyl, etc.).

### Link layer

The link layer acts as a bridge between the physical and the virtual layers: through this layer, the physical layer outputs sensing data to the virtual layer, and the virtual layer can also feedback information to the physical layer. This layer is composed by two interconnected sub-layers:

1. **Infrastructure.** The components of this sub-layer are

- *IoMusT networking and communication:* The infrastructure needs to support synchronous musical interactions within the MM, which pose stringent latency, quality of experience, rate, and reliability requirements [48], along with large quantities of data traffic. Potential solutions are represented by 5G and beyond 5G networks, the deployment of ultra-dense networks in edge networks, and the use of Multi-access Edging Computing (MEC).
- *Data integration and storage:* The MM is expected to produce and use large quantities of data related to musical activities. This data needs to be stored and ideally be interoperable across multiple systems, which requires data integration.
- *Computation:* The MM will support interactions among large numbers of users, especially for real-time applications. This entails the use of powerful Musical XR devices as well as powerful computers. The edge computing paradigm is a promising solution to this issue.

2. **Musical Metaverse Engine.** This sub-layer receives as input the data from the Musical Things used by stakeholders, as well as generates and evolves the multisensory virtual world. The components of this sub-layer are

- *Musical XR:* These technologies include multisensory musical environments, virtual reality musical instruments and any XR-based interface used by musical stakeholders to perform their musical activities.
- *Digital twins:* This technology creates replicas of the real world on the basis of the sensing data received in real-time from the Musical Things in the physical layer. These replicas are displayed via XR technologies and evolve as the real world changes.
- *Artificial intelligence:* This technology can be leveraged to enhance the MM with context-aware and proactive musical services, as well as to improve the user experience, the real-time recognition of musical gestures, and the analysis of multimodal musical content. In particular, the Multi-access Edging Computing (MEC) can be enhanced with artificial intelligence methods, to achieve highly accurate digital twins modeling and continuous model updating with sensing data.
- *Blockchain:* This technology can be used to provide proof-of-ownership of virtual musical goods generated and traded by musical stakeholders, as well as to establish the economic ecosystem within the MM.

### Virtual layer

His layer provides the immersive or augmented experience of the virtual world generated by the metaverse engine. The virtual layer may provide feedback to the physical layer, based on the data received from it via the link layer. The main components of the virtual layer are.

- *Avatars:* Each musical stakeholder has a virtual counterpart in the form of an avatar. Other software agents playing the role of musical stakeholders (e.g., a virtual musician or a virtual audience member) may be represented in the virtual world as avatars.
- *Virtual musical environments:* These environments are the virtual worlds within which musical activities are conducted. They encompass virtual objects, host the avatars, and allow the fulfillment of artistic, pedagogic, social, and economic interactions.
- *Virtual musical goods and services:* This refers to digital content that is generated by the virtual musical services providers as well as services supporting musical activities within the virtual musical environments.

## Opportunities

This section provides an overview of the opportunities that the proposed MM vision may bring. Nevertheless, the MM entails not only opportunities but also challenges for stakeholders, their musical activities and their tools, as well as for the environment. Table 1 provides a summary of the pros and cons of each subject discussed in detail in this section and in Section 5.

**Table 1:** Comparison of opportunities and challenges brought by the Musical Metaverse

	Opportunities	Challenges
Musical activities	• Novel forms of musical expression, pedagogy, interaction with musical content, collaboration, remote participation, and remote control	• Insufficient HW and SW tracking and rendering methods for musical control • Low HW ergonomics for musical uses • Insufficient real-time solutions to interconnect musical stakeholders • Integration of different technologies • Lack of realism of the Musical XR environments • Scalability of MM platforms • Lack of standards and interoperability features • High computing demand to support distributed immersive experiences • Artistic challenges
Stakeholders’ data and social aspects	• New musical communities which are decentralized and self-governed • Higher level of inclusiveness	• Privacy and security issues • Need for ethical design principles • Lack of policies, as well as regulatory and legal frameworks • Potential for psychological and social issues
Monetization	• New forms of revenue streams for artists and audiences • Music distribution without intermediaries	• High computational demand to handle blockchain-based applications • Redefinition of the current monetization schemes based on intermediaries
Environmental sustainability	• Reduced pollution due to travels	• High energy consumption • Hardware and software longevity

### New musical activities

Contemporary musicians need (and historically have always needed) enhanced ways of musical expression and interaction with music and their audience. Meanwhile, today’s audiences seek more engaging musical experiences and a closer interaction with the performers, as is indicated by a growing body of research beyond the metaverse domain [78–80]. The MM is expected to bring unprecedented opportunities for catering to these demands of musicians and audiences. A preliminary proof of this is already visible in the increasing use of the NFTs, which are expected to be even more utilized in the MM. Taken together, the NTF-related trends presented in Section 2.3 are an indication of a profound (and likely lasting) change in how musicians create music and engage with their audiences and fans. Essentially, we are witnessing a new fan–artist relationship, where the fan not only acts as a direct patron, but also can get revenues from the support provided to the artist.

It is plausible to expect that the new technologies at the basis of the MM will spur the emergence of novel forms of musical activities. This has already been proven by the numerous concerts conducted in the past 2 years by different artists (including the renown ones), which took place in immersive virtual environments. Likely in the future, performances in the MM will be an integral part of musicians’ practices and a major source of income. Various VR platforms exist which allow musicians to create virtual environments and perform within them. Notably, some of these platforms (e.g., VRChat, AltspaceVR, Fortnite) have a wide user basis (millions worldwide), who could become the audience of such concerts. Today, we are witnessing the increasing demand of musicians and audiences for networked musical experiences, as evidenced by the COVID-19 pandemic, when music became a positive socio-emotional coping mechanism [81] with 60% of European festivalgoers watching at least one music live stream during lockdown.^4^ The negative impact of the pandemic on the live-music industry has also boosted the use of the metaverse as a place where to conduct live performances.

Whereas at present the degree of realism of connected musical experiences is rather low, the MM vision predicts that in the future compelling collaborative musical activities will be possible. This includes not only live performances, but also recreational music making and teaching/learning experiences. Nevertheless, more than conventional music experiences, the metaverse fosters opportunities for devising completely new ones, which are only possible in virtual worlds. A noticeable example in this space is represented by the 360° experience provided in 2016 by the Philharmonia Orchestra conducted by Esa-Pekka Salonen. Through the app, audience members were empowered with the unprecedented possibility to move on the stage exploring the various sectors of the orchestra, stand near the conductor, see the backstage, or enjoy the performance from the usual seats.

Other opportunities for novel musical activities arise from the use of artificial intelligence techniques [40]. For instance, virtual agents acting as musicians in the MM could interact with real musicians, to co-create music or provide information to them (e.g., in the form of dialogues). In a different vein, XR technologies can support composition in unprecedented ways, leveraging spatial affordances that are only possible in 3D worlds. This is the case for instance of the PatchXR company, whose products allow musicians to build their 3D musical instruments using visual programs that represent the flow of musical events.

Furthermore, the use of digital twins could lead to novel musical processes and services. An example concerns rehearsals or performances involving a remote musician interacting with other co-located musicians. The remote musician could remotely control a specific musical instrument that is present in the room where the other musicians are placed, operating on the digital twin that fully replicates the controls of the physical instrument. A similar use case is reported in the literature for the remote control of analog synthesizers controllable via a web application (see [82]. Along the same lines, remote training or use of musical equipment (e.g., mixers in recording studios) could be performed by leveraging a digital twin to learn or simulate the equipment behavior so the right setup can be identified. Moreover, the exact components of a given musical environment, such as a concert venue (with stage lights, smoke machines, etc.), could be replicated to enable the familiarization with that environment before the musical activity is conducted, such as rehearsals before the concert.

### New musical communities

The metaverse is characterized by XR social environments, which are the result of the convergence of social networks and XR technologies. Such a convergence has also impacted the musical domain. In a social XR environment, users interact with other users through avatars that represent them. The emergence of the MM likely will not replace real-world music-based social relationships with virtual/augmented ones, but rather may generate a new way of how we interact and socialize online. As a new type of social form, the MM includes economic, cultural, and legal systems. These systems, although related to reality, have their own characteristics. It is the exploitation of these characteristics that are peculiar to the XR medium which can lead to the emergence of new social relationships in music contexts.

One of the distinguishing features of the metaverse is community ownership, and this is true also for the MM. Communities in the metaverse may form around a given interest through engagement and collaboration of different users. Such communities are decentralized and self-governed: new designs or services are proposed, subjected to the vote of the community, and selected for the final design on the basis of the highest number of votes. Thanks to this decentralization, many users from the community are responsible for providing the designs or services all users rely on, instead of one central authority (e.g., a single company) controlling what users are allowed to do with their own content and digital properties. These trends are visible for several virtual platforms (e.g., Decentraland, Axie Infinity) specific for gaming rather than music, but it is plausible to expect that community-created 3D worlds for musical activities will emerge, along with the users’ right to own and sell digital music-related items and properties. Relatedly, these trends are an indication that digital creation is more and more democratized. MM users will be empowered with the possibility to create new content in XR environments. This will be accomplished independently from the level of professional expertise in music or XR.

### Monetization

The development of the MM, and the metaverse in general, is still in its infancy. As a consequence, its business model is not mature. Nevertheless, several monetization opportunities are and will be available. The metaverse is expected to be a parallel world having several of the same monetizable aspects as the real world. Moreover, there is the potential for novel forms of revenue streams to arise. Such trends are already visible today for the music sector, as discussed in Sect. 1.2. New business models and forms of monetization have recently emerged encompassing independent musicians, emergent and established bands, as well as musical services providers.

The use cases for the music industry are numerous and include for instance the following:

1. Music will be streamed and concerts will be viewed in the virtual worlds, which offers to the traditional revenue streams a novel ecosystem to monetize (e.g., selling NFTs for tickets of concert taking place in the virtual world);
2. Music will be distributed without intermediaries (evidence of this can be found in various successful platforms such as Audius, Opulous, BitSong, or Royal), and the use of blockchain technology and smart contracts will ensure that musicians get paid fairly and immediately;
3. The fan will directly support the musicians through different means, without passing from the record labels or any third party; these means include for instance buying merchandise in the form of digital assets (e.g., virtual t-shirts) as well as the purchase of the royalties of the musical composition or a digitally signed copy of it (but there is room for devising completely new methods);
4. Digital creators could build their own virtual stages, concert halls, rehearsal rooms, etc. and monetize the usage of those virtual spaces;
5. Musicians can create a membership token that grants special access to a part of the MM (the fans and in particular the super fans are willing to pay for having these peculiar services).

### Benefits for the environment

The realistic interconnection of geographically dispersed musicians and audiences will allow for the remote conduction of different musical activities. By enabling musicians to rehearse remotely instead of commuting to rehearsal rooms, the time and costs for travels will be drastically reduced, and this will result in zeroing any pollution due to travels. The same applies for music teachers and students, but most importantly for audiences, who will be able to experience the sensation of being physically present in the concert venue while being instead placed at their homes.

### Inclusiveness

The MM carries opportunities for disabled musicians and audiences to access musical experiences in unprecedented ways. For instance, concerts in the metaverse could provide easy accessibility to physically disabled audience members, for whom usually it is challenging to attend real-life concerts. New XR-based musical instruments could facilitate the act of playing music for physically disabled musicians. In general, the MM could offer new ways to include such stakeholders, involving them in immersive XR experiences and letting them do remotely things that in real life could not accomplish otherwise.

## Challenges

In this section, we focus on the challenges specific to the actual implementation and functioning of the MM. Other open issues common to the general metaverse field are discussed in other recent reviews (see, e.g., [3, 4, 83] and are also relevant to the MM. Furthermore, as the progress of the MM is strictly linked to that of the underlying hardware and software technologies, the challenges previously identified for the Musical XR field [14], blockchain [84], or networking [47] are also relevant to the MM. This section, however, aims to provide novel and specific contributions about the MM topic as such.

### Technological challenges

#### Interaction with musical content

Hardware and software technologies for composing, playing, performing, teaching, learning, experiencing, and consuming music in the metaverse are not mature yet under different perspectives. First, XR technologies and associated control devices are arguably not lightweight and natural enough, their cost is still not affordable for many, and they typically have a fairly steep learning curve. Second, existing XR devices are not conceived specifically for a musical usage, while music playing is an activity that requires domain-specific tools: for instance, such tools should support a high degree of accuracy in the control of virtual objects producing sounds to properly support the musicians’ expressivity.

Third, latencies between users’ gestures and the corresponding rendered audio-visual stimuli are particularly critical to musical applications. At proprioceptive-visual level, the “motion-to-photon latency” refers to the delay between an action performed by the user and its impact on the scene visually displayed by the XR headset [85]. At proprioceptive-auditory level, the “action-to-sound latency” refers to the delay between an action performed by the user and its impact at auditory level on the sonic environment delivered by headphones or loudspeakers. In the design of digital musical instruments, a generally accepted value for such a latency is 10 ms [86]. Musical XR applications not only should minimize both these latencies, but should also avoid any perceivable latency mismatch between the rendered auditory and visual stimuli. Furthermore, haptic feedback is an important aspect typically missing in musical instruments built with XR technology, which is critical to accurate, real-time control of musical sounds [87].

All such aspects are detrimental not only to the sensation of presence, but also to the experience of controlling the musical sounds. They are hampering a widespread usage of XR technologies by both musicians and audiences, which inevitably has also a negative impact for the MM. Therefore, there is a need for more natural, easier to learn, and more affordable Musical XR devices and software. Importantly, the design of Musical XR applications should consider the different spatial and temporal resolutions of the auditory and visual perceptual systems, and the mechanisms underlying multisensory integration [88].

#### Lack of adequate audio tools

A crucial aspect hindering the development of interactive, distributed interoperable applications such as those envisioned in the MM is the inadequacy of audio tools currently utilized by XR developers. Various XR engines (e.g., Unity3D or Unreal Engine) support audio, as well as there are web APIs such as Web Audio that can be integrated with XR APIs such as Web XR. However, their development level is still largely insufficient for the specific case of the MM.

For instance, current frameworks for multiplayer (e.g., photon engine) support microphones as input and are specifically designed for broadcasting human voices. For the MM case, there is a need for more music-oriented audio streaming. This is particularly challenging for web-based solutions [89]. Moreover, in the MM, there might be scenarios where every note played by a musician needs to be heard by anyone who is connected, or it should be transmitted to a given location at a given time. This is still hard to achieve with current tools and would entail the integration of different technologies such as NMPs systems, which are not primarily conceived for the MM. In addition, web audio and corresponding libraries (e.g., Tonejs) can be easily integrated with frameworks supporting WebXR (such as AFRame, Babylon.js, or 8thWall). However, to date, there is the issue that not all browsers support the use of Web XR (e.g., Apple’s Safari) and, therefore, its integration with web audio.

A complicating factor is represented by the aim of achieving immersive audio experiences, which necessarily entail the use of spatialization technologies that need to be seamlessly integrated [90]. In recent years, many tools have emerged for audio spatialization in the browser [91]. Whereas these endeavors are promising, there is a need to understand which type of spatialization works better and is more suited for multi-user MM. Creating stand-alone applications for most headsets based on Android OS (e.g., Meta Quest, Pico) is still problematic for practitioners and designers. While Unreal, thanks to Metasound (for DSP) and a wide range of built-in synthesis and analysis tools, offers more opportunities to developers to quickly integrate and develop interactive sound systems, Unity3D is way less advanced on this side and it mostly relies on third-party add-ons (e.g., Audiokinetic Wwise) or requires the development and integration of dedicated audio plugins (for example using Faust). However, thanks to its high compatibility with the XR ecosystem, Unity3D still appears to be the preferred solution, especially for small teams and novices. This creates a potential obstacle to the advance of the MM. Not so much effort has been dedicated thus far to integrating and implementing sound-related tools.

In a different vein, sound in an interoperable metaverse might entail that virtual/augmented reality musical instruments should be carried in different places of the metaverse. This opens up a novel design area in terms of usability and distribution of not only visual, but also musical/sonic models. Finally, concerning the sound delivery methods, headphones would be the preferred choice to create immersive experiences that are easily portable. However, to date, a large variety of headsets do not have them or are equipped with poor quality ones.

#### Rendering of musical virtual environments

Current virtual environments designed to host musical activities have different limitations. The first issue is that existing musical virtual environments are still little realistic and too videogame oriented. This constrained situation can work for certain musical activities (e.g., streaming virtual concerts for an audience) while for other activities (e.g., musicians performing together in a virtual environment) a higher degree of realism may be necessary.

Concerning the scalability of multiuser concerts in the metaverse, current visual engines do not support the organization of virtual concerts for a large audience. For example, as of today the platform VRChat allows only a maximum of about 40 users in one room. In addition, current methods to properly recreate the sense of presence in real concerts are insufficient. This includes the integration of the musical sounds generated by the performers with the soundscape resulting from sounds produced by the connected audiences (voices, clapping, etc.) and other background sounds. New techniques are therefore necessary to address such auditory issues. Binaural technologies [92, 93] have the concrete potential to cover a prominent role but have still a marginal utilization in the metaverse, especially in the large platforms mostly used today.

#### Social musical interactions and communication latency

The MM entails strict requirements for a fully immersive experience, large-scale concurrent users, and seamless connectivity. This poses many challenges to networking systems and in particular wireless systems [76], such as ubiquitous connectivity, ultra-low latency, ultra-high capacity and reliability, and strict security. Current methods to musically connect musicians as well as audiences in the metaverse are largely insufficient. End-users need real-time solutions that truly give them the feeling of being together in the same environment, sharing the same musical experience. This is crucial for a successful joint coordination of sounds and movement, and eventually for realizing strong feelings of shared musical experience and sense-making.

The fundamental issue that is necessary to overcome is latency and its variation (jitter), at both auditory and visual level. To play together over the network with stable tempo and in realistic ways, musicians need to communicate their musical signals with latencies below 20–30 ms and with a constant jitter. Academic and industrial research in the field of networked music performances [48] has developed a number of systems to address such issue at auditory level. These systems have encompassed dedicated hardware and software to minimize audio processing latencies as well as codec and networking systems for reducing transmission latencies. On the other hand, relatively little research has been conducted for the low-latency transmission or control of virtual/augmented visual content in metaverse applications, and this is even more true for musical applications. Systems specific for low-latency networked interactions in the metaverse have not been properly designed yet and there is much room for progressing the field [14, 17]. In particular, the most recent studies in this space indicate that while for the acquisition, communication, and display of audio content significant progresses have been made to achieve low latencies (of course compatibly with the physical distance), existing hardware and software methods for the tracking, communication, and rendering of visual XR content are time consuming. Such a gap causes an audio-visual mismatch in the received content that is not tolerable by musicians [51, 52]. This highlights the need to progress the hardware and software methods for the visual tracking, streaming, and rendering of the connected musicians, as well as its integration with the audio content.

#### Integration and standardization

Different technologies contribute to the MM: Musical XR technology provides immersive musical experiences, digital twin technology generates a mirror image of the real world, blockchain technology offers the means to build a monetization system, and 5G/6G infrastructures provide the tools for interconnection. The integration of such diverse technologies is challenging. This is particularly true because most of them are not consolidated yet and are constantly evolving at a steady pace. This may cause issues of fragmentation and compatibility that are non-negligible. Furthermore, metaverses created by different companies cannot typically interact or exchange information/digital assets, given the fact that they are built with different, proprietary and bespoke technologies. Moreover, different currencies/tokens are adopted in different metaverses.

To cope with such issues, there is a need to define and agree on standards for the MM. This will allow developers and users to avoid fragmentation and facilitate interoperability among different MM platforms. However, to date, standardization activities specific to the MM are mostly unrealized.

#### Computing power

Building the MM requires a powerful computing system along with efficient ways to manage the computational power. This is needed to allow a large number of musical stakeholders to interact musically in the MM without experiencing interruptions in their immersive experience [1]. However, existing systems cannot yet meet the intensive computational load requirements of the MM (this is especially true for virtual concerts with a large audience). The number of users (or better, avatars) that can be supported by a given server varies depending on the power of the server and the quality of the software platform handling the shared virtual environment. Nevertheless, the paradigms of cloud computing [94] and edge computing [95] have the potential to address such requirement and, therefore, to become the main infrastructure of the MM. This is in particular relevant for 5G architectures [96, 97].

### Ethical, sustainability, and regulatory challenges

#### Ethics

Notwithstanding the numerous societal, artistic, and economic benefits that MM promises, the degree of immersion in virtual worlds, ubiquitous nature, and increased autonomy of XR products raise concerns about the ethical compliance of the associated services. Ethical and responsible innovation (Van den [98] are crucial aspects to take into account in the design of the MM to ensure that it is socially desirable and undertaken in the public interest. Exploring the ethical implications of the MM must consider principles of responsible practice like the protection of the privacy of users and the protection of the confidentiality of any data collected so that risks to MM participants are eliminated, e.g., the risk to expose personal or sensitive information.

Ethical research in the music technology field has identified various issues that are also very pertinent to the MM, but to the author’s best knowledge, no investigation has been conducted yet on them within MM contexts. For instance, researchers have questioned the practices of music streaming services in monitoring users and inducing behaviors [99] and examined a variety of potential bias in the use of artificial intelligence techniques applied to musical signals [100, 101]. Some have discussed political issues inherent in new music instruments, while others engaged with topics such as gender diversity [102] and accessibility [103]. In a different vein, ethical research in XR has also identified issues that necessarily concern also the MM. In particular, Slater et al. [104] highlight several issues deriving from the increasing “superrealism” of XR experiences, where elements and even experiences in XR may become indistinguishable from reality.

There is a need to follow policies supporting social issues in the MM in order for it to be socially acceptable. A first step could be the precise identification of the major issues (e.g., privacy, transparency, trust, social equality, gender equality, accountability). A second step could consist of the definition of ethical guidelines that can inform design and development of policies in support of the MM. These steps would parallel similar efforts conducted in the related field of IoT [105, 106].

#### Environmental sustainability

Whereas the MM, like the metaverse, to date remains mostly an idea and not an implemented reality, it is paramount to interrogate ourself already now about the potential issues concerning environmental sustainability. Ultimately, these will mostly depend on the choices that will be made by developers and companies about how to implement and run MM platforms, as well as on how musical stakeholders will use them. Recently, there has been an increasing attention devoted by scholars to the negative impacts that music technologies development and use may have on the environment [ 107–109]. Those lines of enquiry are also relevant to the MM.

A first crucial risk concerns the drastic increase of energy consumption, with the consequent influx of greenhouse gasses emitted. XR technology and data centers use artificial intelligence methods and cloud services, which require quite large amounts of energy. A lot of energy will be also needed to store a great quantity of data concerning musical stakeholders and the virtual worlds, as well as to power severs to process such data. As more musical events and activities take place online, more energy will be needed.

Relatedly, the extensive use of blockchain technologies and in particular NFTs, which are notoriously energy hungry, will lead to a high energy usage and consequently to a negative environmental impact [110, 111]. Notably, the attention and sensitivity toward these topics has started to emerge in the artistic community. For instance, the digital artist Joanie Lemercier cancelled the NFT production and sale of six artworks after calculations revealed that the process for NFT creation, use, and verification would use in just 10 s the same amount of electricity consumed in his studio in 2 years.^5^

Another major issue concerns the longevity of the hardware and software tools used in the MM (an aspect investigated also in other domains of music technology; see, e.g., [112]. It is plausible to expect that the continuous advancements of Musical XR technologies will encourage MM users to buy new hardware due to the setting of new hardware requirements to interact in the virtual worlds (e.g., the need of novel high-end GPUs or new HMD with higher resolution). This necessarily will translate in an influx in e-waste, which consequently will contribute to polluting the soil, groundwater, and landfills.

#### Privacy and security

Privacy plays a crucial role in shaping the MM. However, only a few studies have investigated privacy issues in Musical XR contexts [113]. How exactly to ensure privacy in Musical XR remains an open question, despite the fact that it is potentially as important as it is in reality [114]. Given the pervasive presence of the metaverse, transparent privacy mechanisms will have to be implemented on a diverse range of Musical XR products and services as well as on the platforms that support them. The application of privacy-preserving schemes is much easier in conventional social networks compared to the metaverse, as users can decide with whom to share their social media content. In the metaverse such privacy control is not possible, as users cannot change the properties of the virtual environment in which they are placed [115].

It is necessary to address issues of data ownership in order to ensure that MM users feel comfortable when participating in musical activities. MM users must be assured that their data will not be used without their consent. The definition of privacy policies is one approach to ensure the privacy of information. Hardware and software devices for the MM can be equipped with machine-readable privacy policies, so that when they come into contact, they can each check the other’s privacy policy for compatibility before communicating [116]. Based on this, it is therefore crucial that Musical XR manufacturers and service providers adopt a “privacy by design” approach [117] as well as incorporate privacy impact assessments into the design stage of Musical XR products and MM platforms.

Security is another element that has been scarcely addressed in Musical XR research. The MM should prevent sessions from being attacked. Moreover, identity thefts or fake identities represent a major risk for users, especially considering the use of advanced superrealism techniques capable of creating in the virtual world false copies of real people, including their physical attributes and their personality [104].

#### Law and policy

The MM will inevitably induce new legal challenges that must be addressed through a shared effort involving all interested parties. A new legal environment specific to the MM needs to be developed. For instance, there is a need to develop renewed legal approaches for the protection of privacy and copyright in the different music sectors as well as hardware and software techniques [118]. The current copyright and intellectual property laws, which enable owners of musical content to control the reproduction, distribution, and public performance of their works, need to be adapted to future MM scenarios [119].

New policies and regulations are required to address any relevant policy gaps to support the new musical ecosystems of the MM. New research efforts should focus on the definition governance guidelines and regulatory options to support the responsible development and use of MM products. Digital piracy over the Internet is still a problem in the music industry and it is plausible to expect that this issue will persist within the MM, although the adoption of NFT and blockchain technologies is expected to drastically reduce such an issue.

A relevant issue concerns the territoriality of the regulations which the companies offering MM services have to adhere to. For instance, the GDPR holds for companies based in Europe, but the musical virtual worlds created by such companies can achieve worldwide proportions. This may cause issues with extra-EU regulations, and therefore adaptation schemes must be set in place by these companies for the different countries in which they operate.

#### Psychological and social issues

The MM could entail issues at psychological and social level. Firstly, there is a risk for user addictions, an effect demonstrated for other related media and highly relevant to the metaverse [120]. Despite music is an activity that typically enriches our lives at multiple levels, the excessive use of the MM could lead to physical, social, and mental disorders. Social isolation is another concrete issue, where MM users would tend to avoid interactions with other people in real life. Relatedly, MM users could exhibit issues of preference for virtual social interactions compared to real ones. Notably, superrealism may let users experience musical activities so highly resembling the real-world ones that the issues above could be further exacerbated [104]. In a separate vein, the MM could be potentially affected by cyberbullying. Dedicated software methods should be devised to detect such misbehaviors in the same way it occurs for other media [121].

Another line of enquiry concerns the prolonged use of the MM. To date, the attention devoted by researchers to the investigation of how performing musical activities using XR technologies compare with the conduction of the same activities in the real world remains limited. Only a few studies have been conducted in this space for non-musical domains [122, 123], and results point to the incapability of current XR technologies to provide a seamless user experience that would support users for long working sessions. In general, new investigations, especially using in-the-wild methods, should be conducted to understand users’ behaviors in the MM. Moreover, there is a need for technical and non-technical methods for prevention of the disorders possibly caused by erroneous uses of the MM.

### Artistic challenges

Likely, the main artistic challenge relates to the adoption of the MM by the artists. This will be inevitably linked to the social acceptability of the hardware and software tools, along with their effectiveness to support the artists’ creative processes and musical activities.

Exploiting the possibilities offered by the MM entails a rethinking of conventional musical activities and practices. To progress the music field toward new directions, composers and performers should focus on the aspects that are peculiar to the MM, and all its underlying technologies. For instance, they will need to consider the distributed nature of musicians and audiences within MM ecosystems, along with the multimodality of the musical content. This is challenging and spurs the conduction of a whole new kind of artistic research in this unprecedented space.

Music pedagogy could also be benefitted by the MM affordances, but applications in this space are still in their infancy [14]. There is a need for novel systems and human–computer interaction investigations that could exploit the unique opportunities of the MM for devising new teaching and learning methods.

On the other hand, audiences could also leverage the MM opportunities and change their way of intending their interaction with music and their favorite artists. For instance, audiences could participate to the creation process of an artist’s music, or to its surrounding decision processes through community voting schemes, typically reserved to fans with special access (e.g., granted via NFTs). This is essentially a new form of making art, which subvert the conventional roles of the artist fully in charge of the creative process and of a passive audience.

In general, the MM could be seen as a new form of society, and this is expected to give rise to new cultural forms. Music, after all, has always accompanied humanity in its evolution, including societal and cultural changes. The MM could be a new space for artists to promote political ideas through their music. However, this could also generate censorship issues by authorities.

## Conclusions

In this paper, we aimed to investigate not only the potentials of computational music in the dawn of the metaverse era, but also the related concerns. The metaverse is expected to profoundly impact the organization and functioning of our society through the integration of XR, blockchain, 5G/6G, digital twins, and artificial intelligence. This is also true for musical activities and social relationships based on them.

The MM may foster several new opportunities for the music industry and the musical domain at large, paving the way to new services and applications capable of exploiting the interconnection of the digital and physical realms. The music industry is increasingly becoming one of the biggest investors in the metaverse and the MM has the concrete potential to disrupt the music industry sector as we know it today. On the one hand, the MM can transform how musicians earn a living through the use of digital property rights; on the other hand, fans are offered new ways to connect with the music, their favorite artists, and each other. The accomplishment of the MM vision will undoubtedly take many years of development, but has the potential to result in an enriched range of musical interaction modalities that ultimately are expected to greatly benefit a large variety of musical stakeholders. This potential, however, needs to be accomplished on the basis of a solid understanding of the associated ethical concerns, which are yet to be defined.

Ideally, the development of the MM (including new services, hardware and software tools, and Musical XR techniques) should be intended as an interdisciplinary endeavor focused on addressing, seamlessly and simultaneously, technological, artistic, perceptual, economic, ethical, and social aspects related to the conduction of musical activities in this new space. Moreover, it should be a collaborative effort which encompasses the voices of all the various stakeholders involved. Of course, in part the responsibility for the creation of socially and environmentally acceptable MM platforms will depend on the companies’ choices about how to develop them. But in part, this responsibility will necessarily depend also on how the end users will use such platforms.

The MM is rapidly growing in artistic, academic, and industrial settings, as testified by new musical activities, products, services, the increasing number of publications from various academic communities (e.g., NIME, Gaming, HCI, Internet of Sounds), as well as talks and tutorials at prestigious conferences and music fairs. Since the metaverse is emerging, now is the best time to establish rules for the MM and associated Musical XR technologies along with policies for the interactions of stakeholders.

As of today, we do not know enough about the impact of metaverse technologies on music and musical stakeholders. We also lack the language and the metrics to discuss, evaluate, and contribute to a critique of these new technologies. Much work remains to be done in this space at technological, artistic, economic, ethical, and policy levels. For the MM to be safely adopted by end users, a number of technical, artistic, personal data-related, and regulatory challenges need to be addressed. Moreover, it is important to address issues of energy consumption and eco-compatibility of manufacturing materials as well as stimulate holistic research that takes environmental goals into consideration. Therefore, this paper calls for more discussions and technical endeavors within Musical XR-related communities, so for the MM to be built in responsible ways.

## Footnotes

1 Video available at: https://youtu.be/OB57zA5MB7o.

2 https://youtu.be/Uvufun6xer8.

3 https://www.ibm.com/topics/what-is-a-digital-twin.

4 https://www.statista.com/statistics/1121434/live-music-streams-coronavirus-lockdown-europe/.

5 https://joanielemercier.com/the-problem-of-cryptoart/.
